# Supplementary figures and images for: Dmrt2 regulates sex-biased neuronal development in the cingulate cortex
Source: Cell Mol Life Sci. 2025 Oct 30;82(1):376. doi: 10.1007/s00018-025-05851-1 (PMC12575903; doi:10.1007/s00018-025-05851-1)

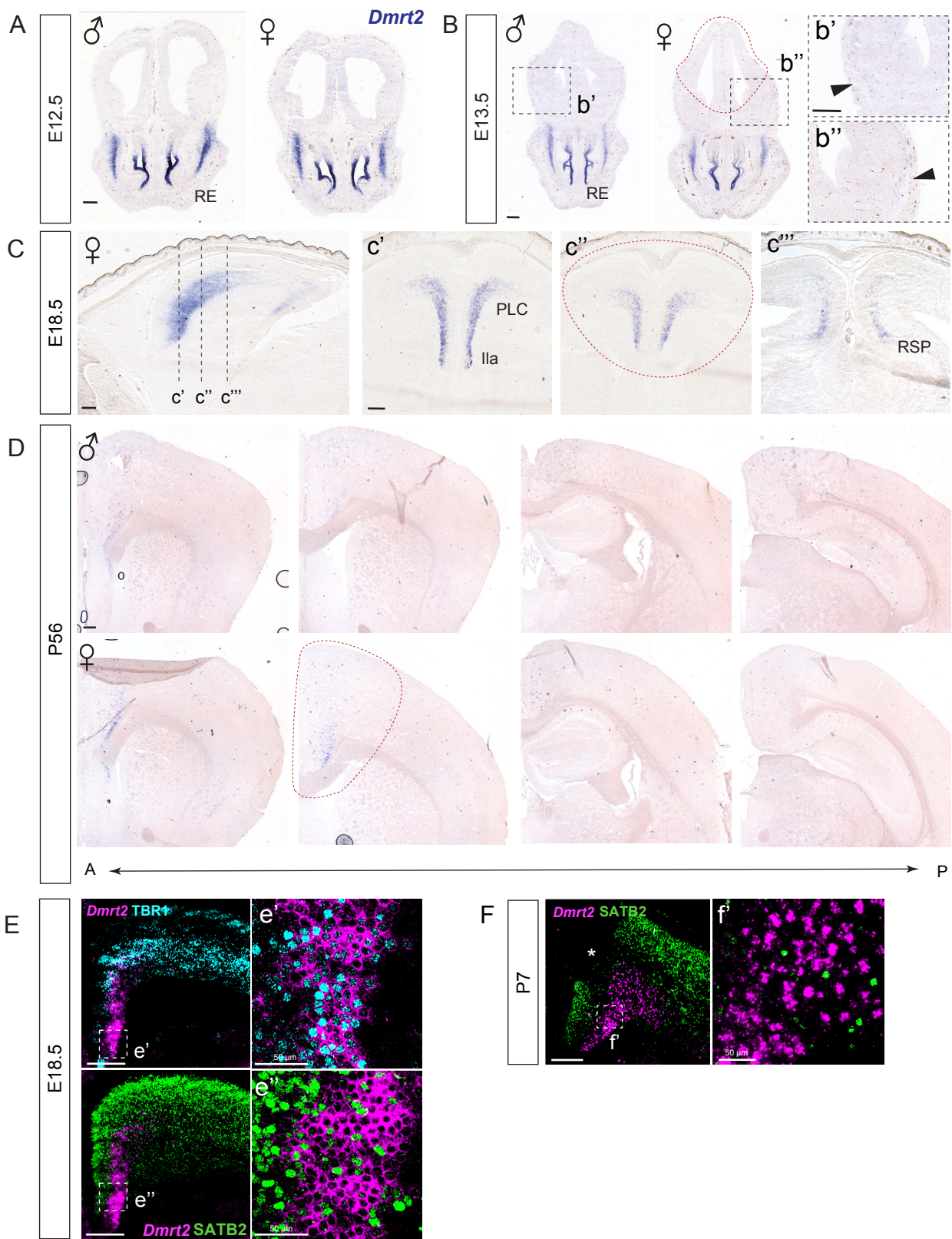

Supplement: Supplementary file 1 — Supplementary file1 (PDF 1932 KB) [file 18_2025_5851_MOESM1_ESM.pdf]

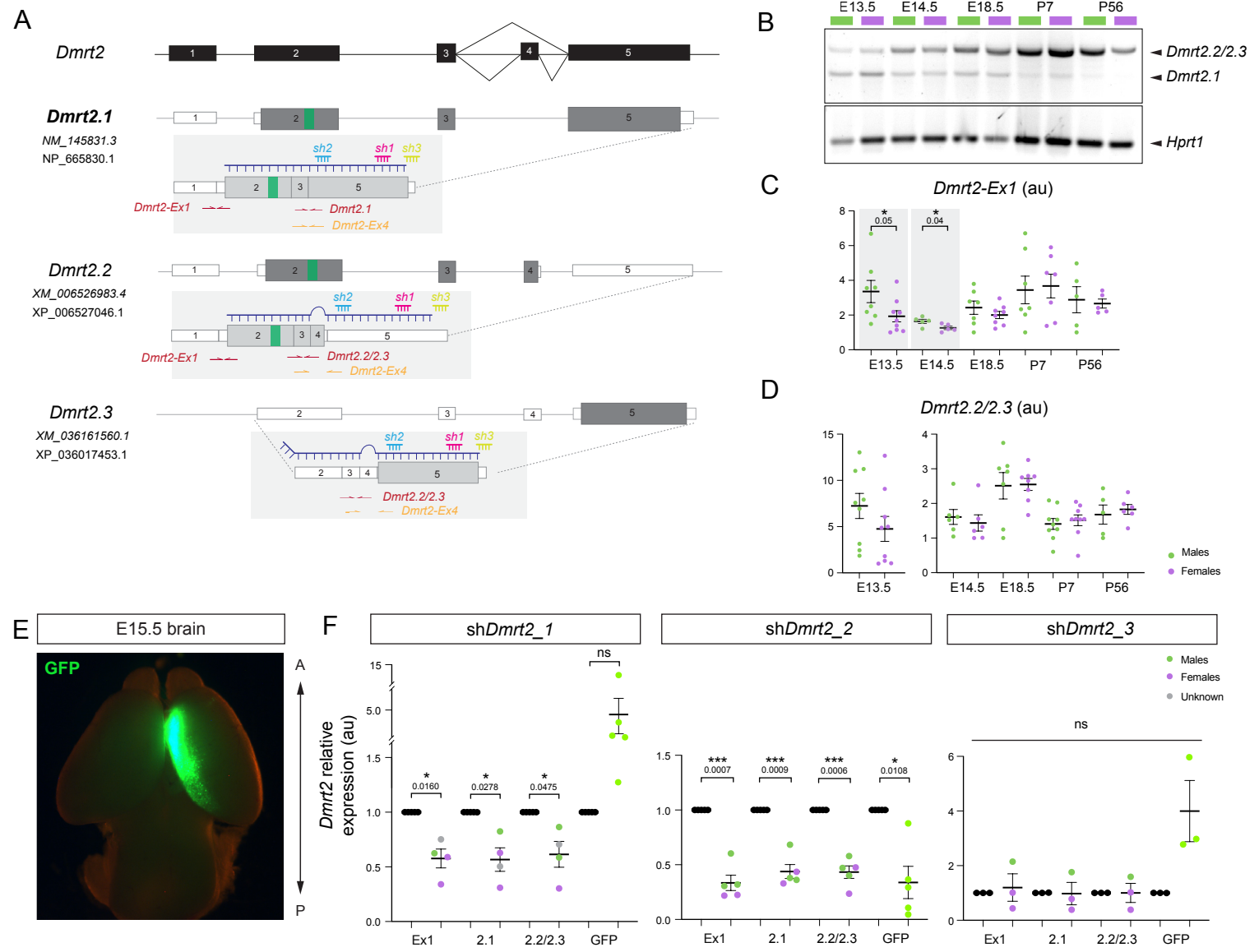

Supplement: Supplementary file 2 — Supplementary file2 (PDF 262 KB) [file 18_2025_5851_MOESM2_ESM.pdf]

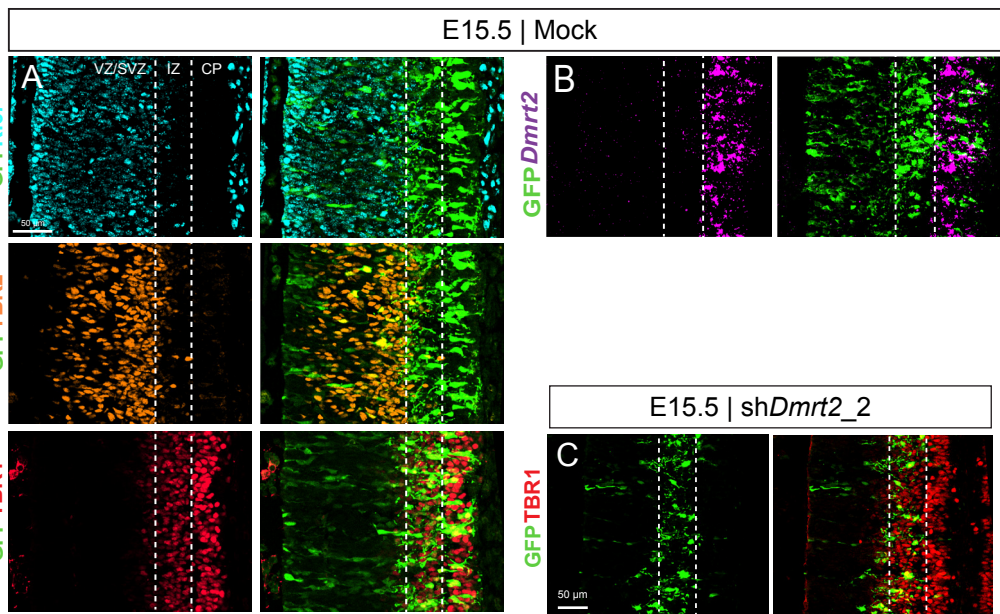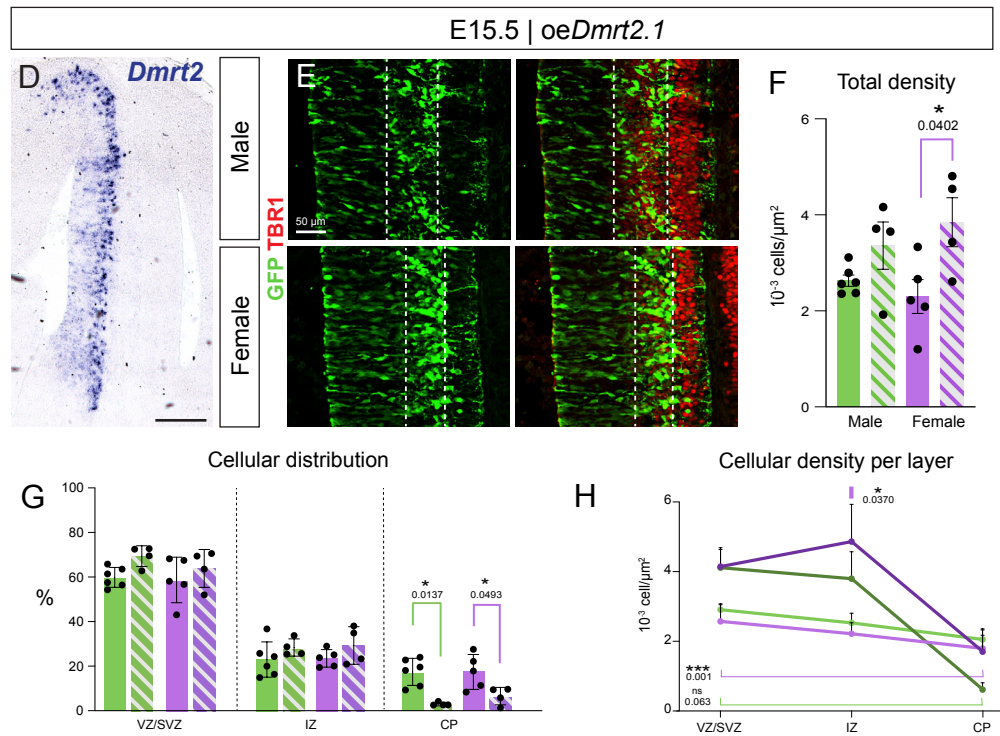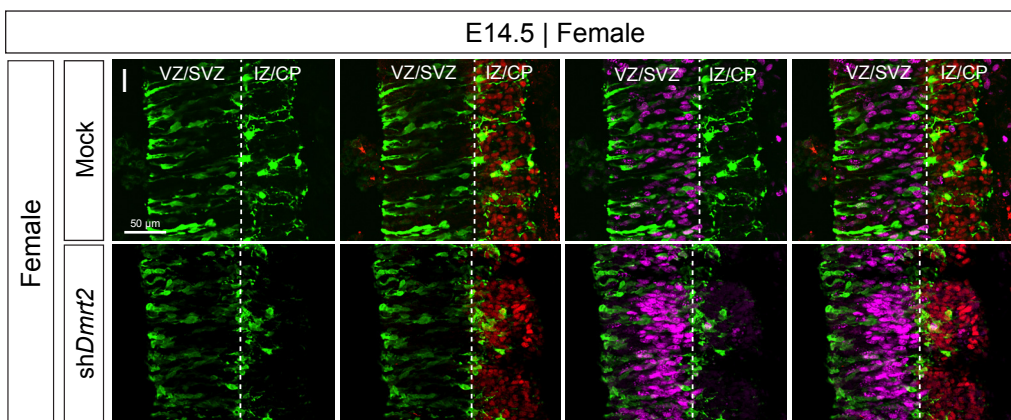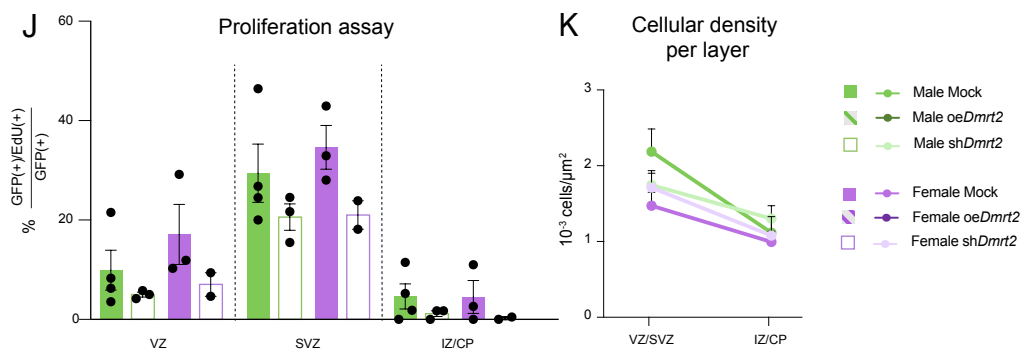

Supplement: Supplementary file 3 — Supplementary file3 (PDF 1975 KB) [file 18_2025_5851_MOESM3_ESM.pdf]

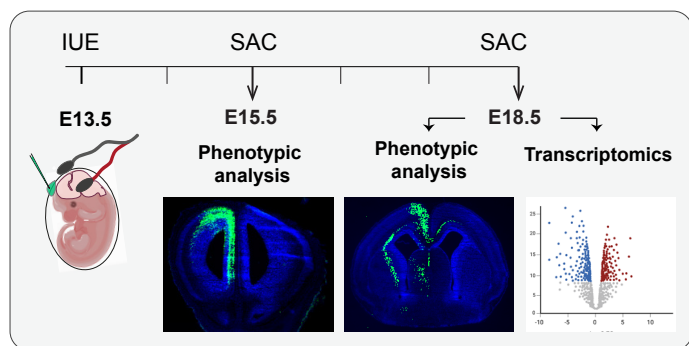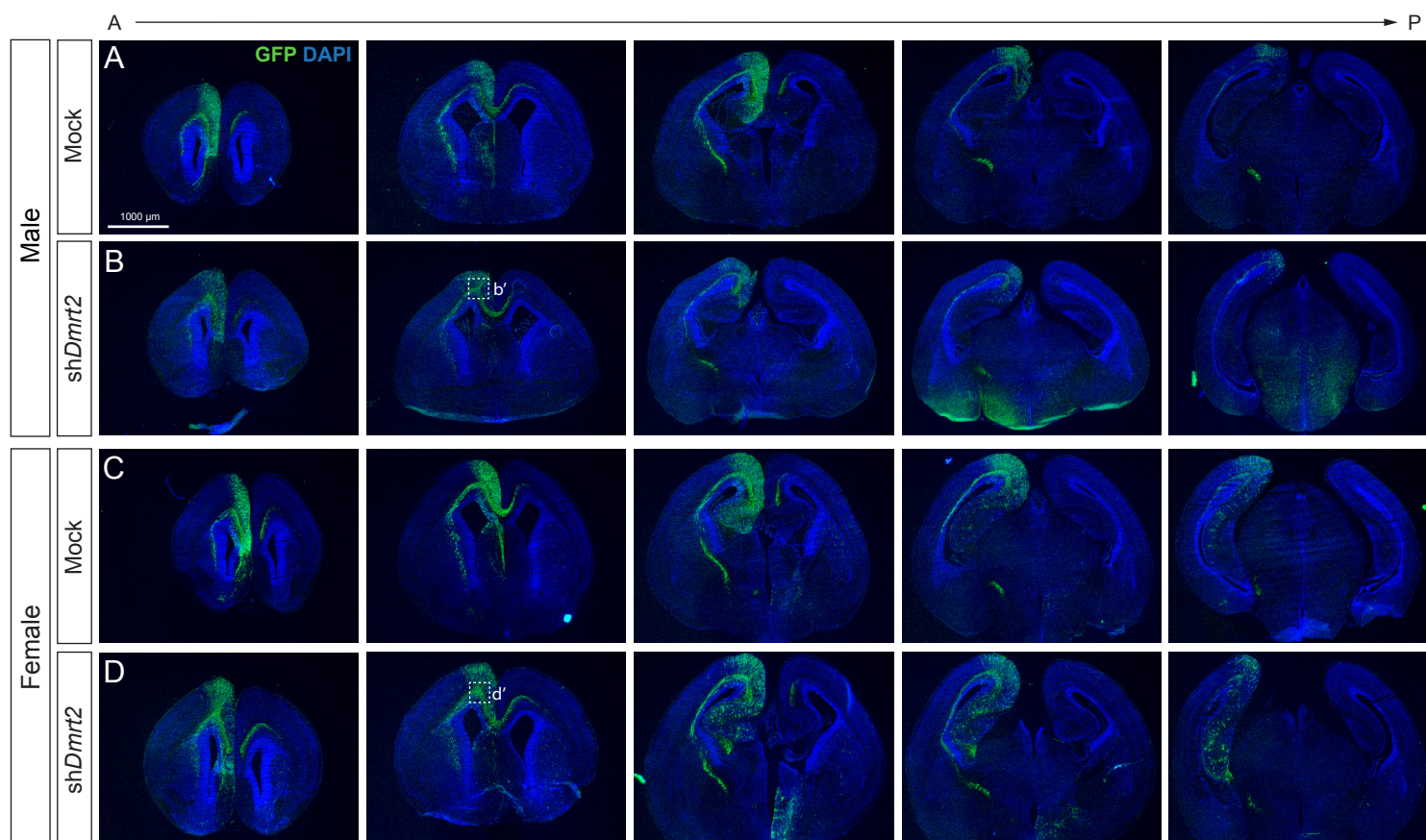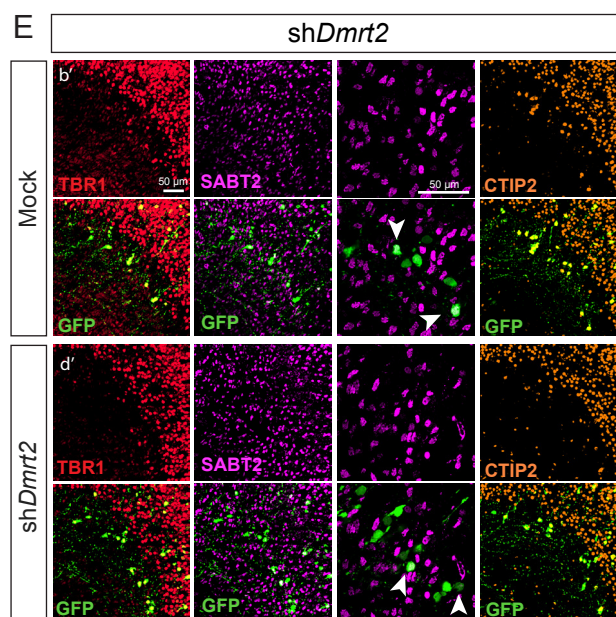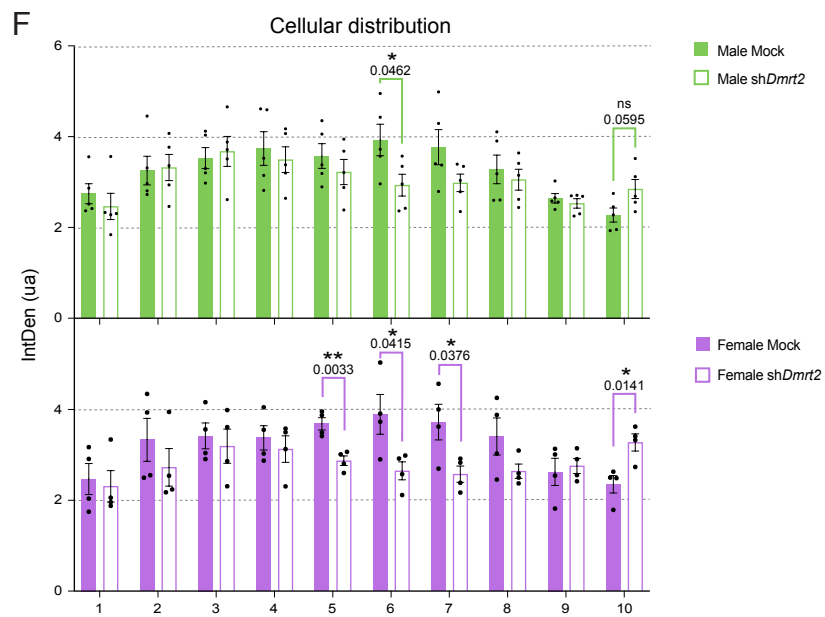

Supplement: Supplementary file 4 — Supplementary file4 (PDF 2208 KB) [file 18_2025_5851_MOESM4_ESM.pdf]

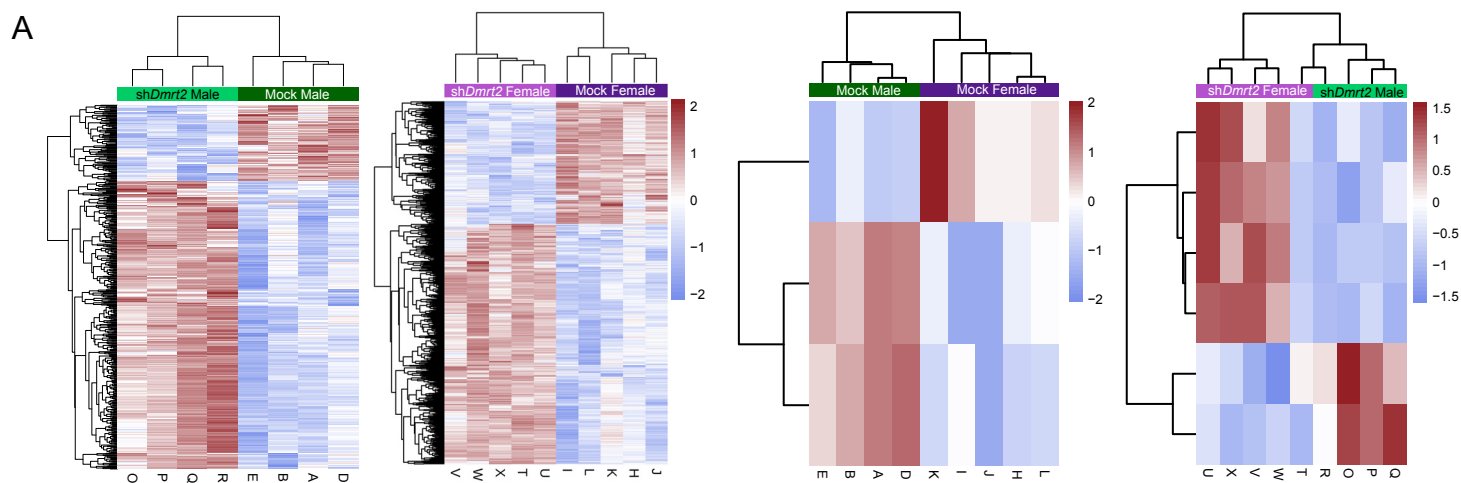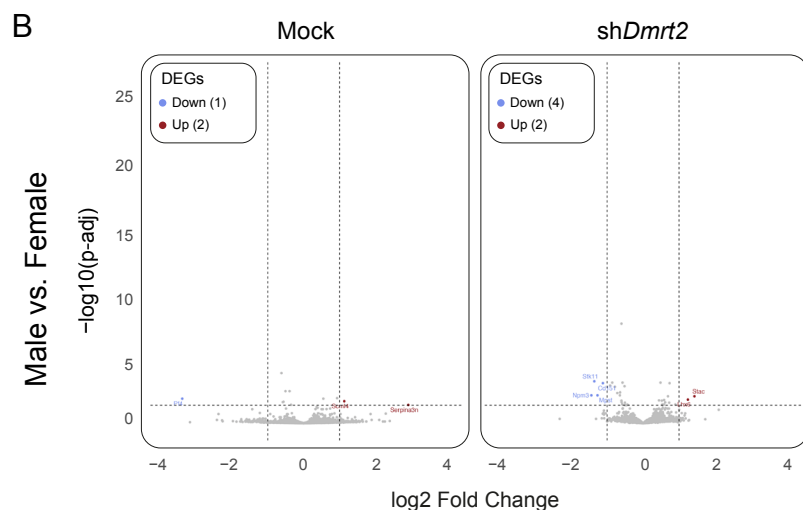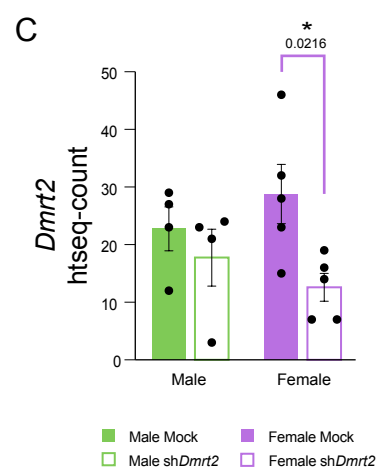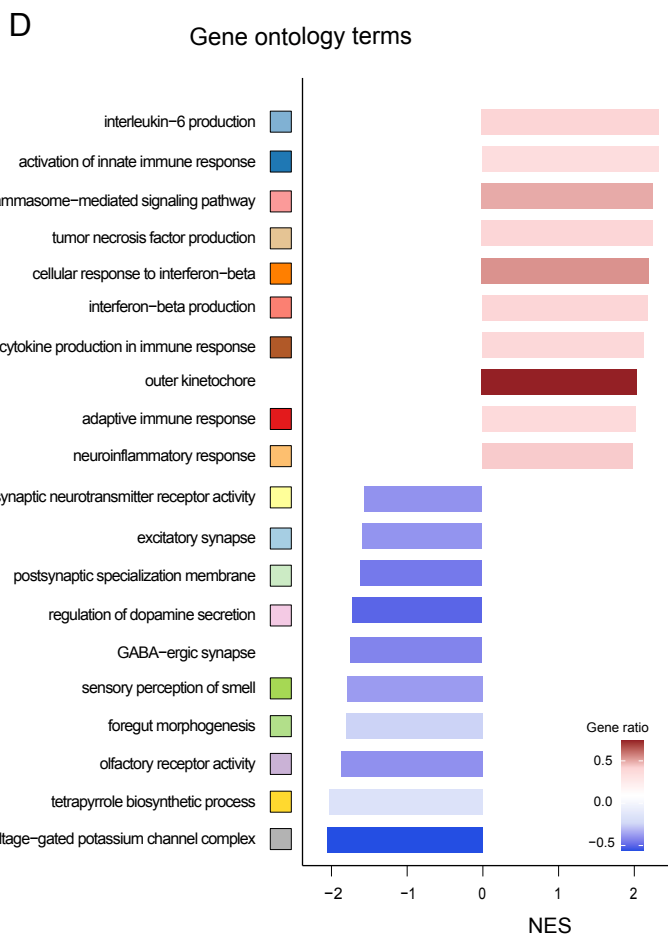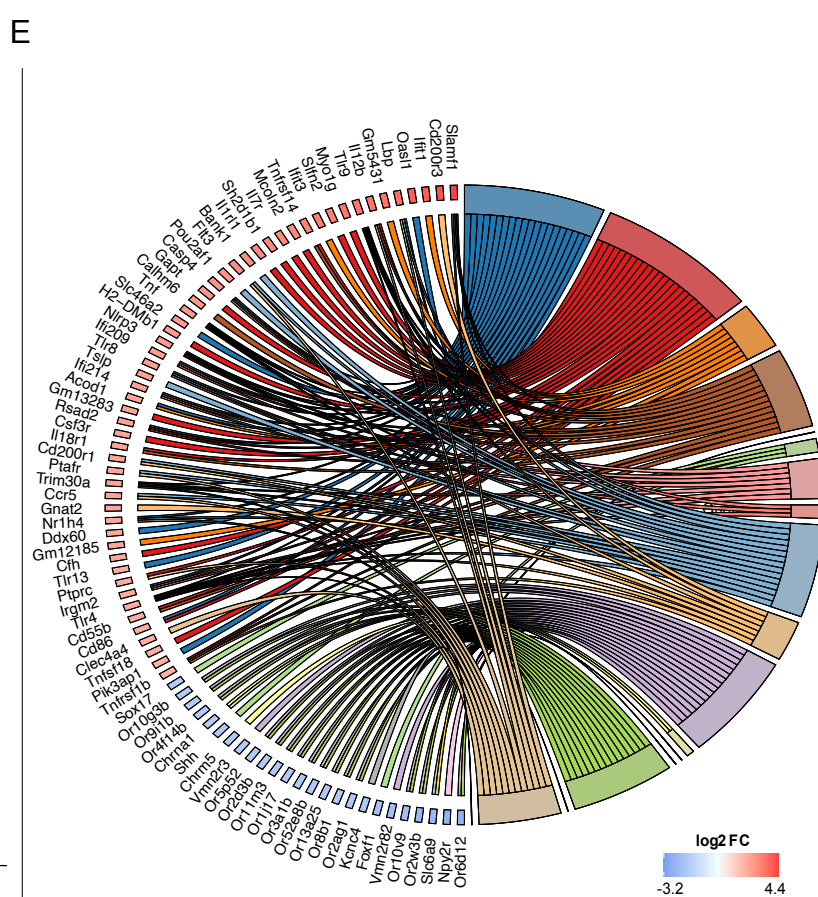

Supplement: Supplementary file 5 — (PDF 754 KB) [file 18_2025_5851_MOESM5_ESM.pdf]
