# Supplementary material for: Dmrt2 regulates sex-biased neuronal development in the cingulate cortex
Source: Cell Mol Life Sci. 2025 Oct 30;82(1):376. doi: 10.1007/s00018-025-05851-1 (PMC12575903; doi:10.1007/s00018-025-05851-1)
Supplement: Supplementary file 6 — Supplementary file6 (PDF 67 KB) [file 18_2025_5851_MOESM6_ESM.pdf]

Supplementary table 1. Aberrant phenotypes in male brains of sh*Dmrt2* and mock electroporated samples at E18.5.

Male Mock							
	**S83**	**S84**	**S97**	**S195**	**S199**		**Mean ± SEM**
**IUE GFP(+) area (μm^2)**	36.43	42.50	40.23	35.08	46.56		40.16 ± 2.08
**IUE GFP(+) fluorescence intensity (au) [(a)]**	125.47	144.25	109.55	116.58	158.34		130.84 ± 9.00
**GFP gaps [(b)]**	|	|	|	|	|		No
**Migratory defect [(c)]**	|	|	|	|	|		No
Defasciculation	|	|	|	|	|		No
VZ GFP(+) cells	++	++	+	+++	+++		Yes
**Male sh*Dmrt2***							
	**S90**	**S100**	**S102**	**S164**	**S165**		**Mean ± SEM**
**IUE GFP(+) area (μm^2)**	37.93	16.87	24.29	34.29	39		30.48 ± 4.28
**IUE GFP(+) fluorescence intensity (au) [(a)]**	94.55	57.44	64.21	87.36	114.12		83.54 ± 10.31
**GFP gaps [(b)]**	++	+	++	+	++		Yes
**Migratory defect [(c)]**	2.25	2.33	4.00	2.33	8.75		3.93 ± 1.25
Defasciculation	+++	++	++	+++	+++		Yes
VZ GFP(+) cells	- - -	- - -	- -	- - -	- -		No

[(a)] Fluorescence intensity of the electroporated area (**Figure 2A-D**) was measured by Integrated Density (area x mean gray value).
[(b)] All gaps are located at the medial cingulate cortex.
[(c)] Number of cells found at the corpus callosum.
(|) No phenotype
(+) Ectopic cells or branching (color intensity increases with phenotype)
(-) Absence of cells (color intensity increases with phenotype)
