# Supplementary material for: Dmrt2 regulates sex-biased neuronal development in the cingulate cortex
Source: Cell Mol Life Sci. 2025 Oct 30;82(1):376. doi: 10.1007/s00018-025-05851-1 (PMC12575903; doi:10.1007/s00018-025-05851-1)
Supplement: Supplementary file 7 — Supplementary file7 (PDF 61 KB) [file 18_2025_5851_MOESM7_ESM.pdf]

Supplementary table 2. Aberrant phenotypes in female brains of sh*Dmrt2* and mock electroporated samples at E18.5.

**Female Mock**							
	**S86**	**S98**	**S99**	**S198**	**S200**		**Mean ± SEM**
**IUE GFP(+) area (μm^2)**	40.56	41.85	44.42	40.56	41.09		41.70 ± 0.72
**IUE GFP(+) fluorescence intensity (au)** [(a)]	99.07	209.82	135.11	139.64	136.24		143.98 ± 18.04
**GFP gaps** [(b)]	|	|	|	|	|		No
**Migratory defect** [(c)]	+	|	+	|	|		No
Defasciculation	|	|	|	|	|		No
VZ GFP(+) cells	+++	+	+++	+++	+++		Yes
**Female sh*Dmrt2***							
	**S89**	**S93**	**S166**	**S167**	**S168**		**Mean ± SEM**
**IUE GFP(+) area (μm^2)**	50.91	45.89	40.47	53.82	38.99		46.02 ± 2.87
**IUE GFP(+) fluorescence intensity (au)** [(a)]	131.63	96.90	147.66	130.22	124.26		126.13 ± 8.27
**GFP gaps** [(b)]	+	++	+	+	+		Yes
**Migratory defect** [(c)]	8.00	7.33	28.33	10.33	18.33		14.46 ± 3.98
Defasciculation	+++	+++	+	++	+++		Yes
VZ GFP(+) cells	- - -	- - -	- -	- - -	- - -		No

[(a)] Fluorescence intensity of the electroporated area (**Figure 2A-D**) was measured by Integrated Density (area x mean gray value).
[(b)] All gaps are located at the medial cingulate cortex.
[(c)] Number of cells found at the corpus callosum.
(|) No phenotype
(+) Ectopic cells or branching (color intensity increases with phenotype)
(-) Absence of cells (color intensity increases with phenotype)
